# Molecular Detection of *Anaplasma phagocytophilum* in Small Mammals and Infesting Ticks in Laikipia County, Kenya

**DOI:** 10.1155/2024/5575162

**Published:** 2024-05-09

**Authors:** Erick Titus Mosha, Joseph K. N. Kuria, Moses Otiende, Isaac Lekolool

**Affiliations:** ^1^Department of Veterinary Pathology, Microbiology and Parasitology, Faculty of Veterinary Medicine, University of Nairobi, Nairobi, Kenya; ^2^Forensic Laboratory, Kenya Wildlife Service (KWS), Nairobi, Kenya

## Abstract

Anaplasmosis is a set of disease conditions of various mammals caused by bacteria species of the genus *Anaplasma*. These are sub-microscopic, Gram-negative, obligate intracellular pathogens that infect both vertebrate and invertebrate hosts. Significant species that infect domestic and wildlife animals include *Anaplasma marginale, Anaplasma ovis*, *Anaplasma mesaeterum, Anaplasma platys*, and *Anaplasma phagocytophilum*. Although *A*. *phagocytophilum* has a widespread distribution, there are only a few epidemiological reports from sub-Saharan Africa. This study focused on molecular detection and characterization of *A. phagocytophilum* in small mammals and their infesting ticks in Laikipia County, Kenya. A total of 385 blood and 84 tick archival samples from small mammals (155 females and 230 males) were analyzed. The blood samples were subjected to a nested PCR-HRM melt analysis using species-specific primers to amplify the 16S ribosomal RNA genes. The ticks were also subjected to nested PCR-HRM involving 16S rRNA gene primers. *Anaplasma phagocytophilum* DNA was detected in 19 out of 385 samples using species-specific 16S rRNA gene primers giving a prevalence of 4.9% for *A. phagocytophilum*. Analysis of the tick's samples using 16S rRNA gene species-specific primers also detected *A. phagocytophilum* in 3 samples from *Haemaphysalis leachi* ticks (3/84) equivalent to prevalence of 3.6%. Sequencing of 16S rRNA PCR products confirmed *A. phagocytophilum* in small mammals and ticks' samples. Phylogenetic analysis of the haplotype from this study demonstrated a close ancestral link with strains from *Canis lupus familiaris, Alces alces, Apodemus agrarius*, and ticks (*Haemaphysalis longicornis*) reported in Europe, China, and Africa. Comparison was also made with a known pathogenic *A*. *phagocytophilum* variant HA and a nonpathogenic variant 1 that were clustered into a distinctive clade different form haplotypes detected in this study. All the haplotype sequences for *A. phagocytophilum* from this study were submitted and registered in GenBank under the accession numbers OQ308965–OQ308976. Our study shows that small mammals and their associated ticks harbor *A. phagocytophilum*. The vector competence for *H*. *leachi* in *A. phagocytophilum* transmission should further be investigated.

## 1. Introduction

*Anaplasma phagocytophilum* is a Gram-negative, obligate intracellular bacterium that causes anaplasmosis in humans and other mammals such as domestic and wildlife ruminants [1]. *A. phagocytophilum* infects neutrophils causing human granulocytic anaplasmosis and anaplasmosis in ruminants, horses, dogs, and cats [2, 3]. *A. phagocytophilum* was previously known as *Ehrlichia equi* or *Ehrlichia phagocytophila* and later recategorized as *Alphaproteobacteria* in the family Anaplasmataceae [4].

*Anaplasma phagocytophilum* has a wide host range and complex reservoir hosts especially in rural and pastoral communities which have a shared ecosystem between humans, livestock, and wildlife [5, 6]. In addition to the *A. phagocytophilum* livestock-human cycle, there is evidence of the bacterium circulating in wildlife vertebrates and reports show that small mammals such as mice, wood rats, and birds can harbor *A. phagocytophilum* without manifesting clinical signs [7–10]. These small mammals are reservoir hosts for many tick-borne pathogens as well as feeding hosts for various stages of tick vectors [11].

*Anaplasma phagocytophilum* is transmitted mainly through bites from ticks in the *Ixodes* spp. group although other species from the genera *Rhipicephalus* [12], *Dermacentor* [13], *Haemaphysalis* [14], and *Hyalomma* [15, 16] have also been documented to harbor and maintain *A. phagocytophilum*. Transmission of *A. phagocytophilum* can also occur through direct inoculation of infected blood into susceptible hosts by use of contaminated instruments [1, 17]. Nevertheless, most human infections occur through tick bites from infected ticks and people working in tick infested areas are at high risk of contracting these zoonotic pathogens [18]. This study aimed at evaluating the presence of *A*. *phagocytophilum* circulating in small mammals and the associated ticks. The generated results will complement the available information on epidemiology of *A. phagocytophilum*, thus creating awareness for policy formulation and control strategies.

## 2. Materials and Methods

### 2.1. Ethical Consideration

This research utilized samples from a study that was reviewed and approved by the Institute of Primate Research (IPR) under the research clearance number ISERC/11/2019.

### 2.2. Study Area and Design

**Figure 1:**
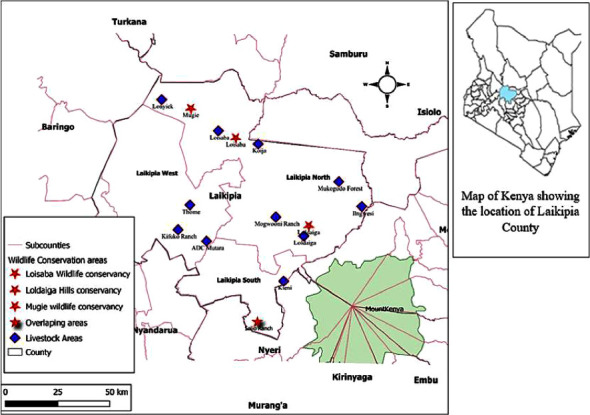
Map of Laikipia County with the areas selected for sample collection marked by stars and diamonds.

The research project was a cross-sectional study utilizing samples collected between 2018 and 2020. Laikipia County is situated in the Great Rift Valley between latitudes 0°18″ South and 0°51″ North and between longitudes 36°11″ and 37°24″ East, covering an area of 9,462 kilometre square. The ecology is classified as semiarid to arid zone with a yearly rainfall of about 300 to 900 mm divided into two minor and major rain seasons [19]. This type of climate favors a savannah type of grassland comprised of a vast grassland and open woodland, coarse thicket thorns, and *Acacia* trees conducive for a wide variety of animals and pastoralist type of economic activity [20]. The area is occupied by a significant number of pastoral settlements with free-roaming small mammal (rodent) species. Samples were collected in livestock areas, wildlife areas, and areas overlapping the livestock and wildlife areas as illustrated in Figure 1.

### 2.3. Sample Size

The study involved analysis of 385 blood and 84 tick samples from small wildlife mammals as illustrated in Table 1. The collection of these samples involved the capture and release method using various traps such as baited traps, Sherman live traps, tomahawk™ traps, and pitfall traps. Blood samples were preserved on FTA™ cards, whereas tick samples were preserved in ethanol.

### 2.4. DNA Extraction

DNA extraction from blood samples involved punching a 3 mm diameter disc from the FTA™ cards. Individual ticks were removed from the ethanol preservative, washed twice using 1x PBS, and crushed into a homogenate in 50 *μ*L of 1x PBS. DNA from both blood and tick samples was extracted using the QIAamp Blood and Tissue Kits (QIAGEN, Germany), following manufacturer's instructions. Concentration of the extracted DNA was evaluated by BioTekfi Powerwave XS2 microplate spectrophotometer after calibration with 1.5 *μ*l PCR water and later stored at −80°C until required for amplification.

### 2.5. PCR-High Resolution Melting Analysis (HRM) of *Anaplasma phagocytophilum*

The extracted DNA was analyzed using nested PCR-High Resolution Melting analysis (HRM) [21], targeting the 16S ribosomal RNA (16S gene) following conditions and procedures illustrated by Massung et al. [22]. The primary amplification cycle involved primer sets CACATGCAAGTCGAACGGATTATTC and TTCCGTTAAGAAGGATCTAATCTCC (ge3a/ge10r) while the secondary amplification used primer sets AACGGATTATTCTTTATAGCTTGCT and GGCAGTATTAAAAGCAGCTCCAGG (ge9f/ge2).

The primary amplification reactions were carried in an Eppendorf T100 thermal cycler (Bio-Rad) at a volume of 25 *μ*L comprised of 1 *μ*l of 10pmol both forward and reverse primers, 12.5 *μ*l OneTaq1 2X Quick-Load Master mix topped up with 9.5 *μ*l DNase/RNase-Free PCR-grade water, and 2 *μ*l of the extracted DNA. The secondary amplification (PCR-HRM) was conducted in Rotor-gene Q thermocycler (QIAGEN, Heidelberg, Germany), with total reaction volume of 15 *μ*l consisting of 0.5 *μ*l each of the forward and reverse primers, 2.0 *μ*l of the primary PCR product, 8 *μ*l of sterile PCR water, and 4 *μ*l of 4 *μ*l 5x HOT FIREPol® EvaGreen® HRM master mix.

### 2.6. DNA Sequencing, Phylogenetic Analysis, and Data Analysis

Positive PCR products were purified and sequenced, and the chromatograms for the forward and reverse sequences were assembled and verified using Biotech Sequencer. The consensus sequences were aligned using MUSCLE v. 3.8.31 [23] algorithm in Molecular Evolutionary Genetics Analysis (MEGA 11) software. Sequences obtained in this study were compared for highest similarity with sequences archived in the GenBank using BLASTn algorithm in NCBI (Bethesda, MD, USA). Representative Sequences of known *A. phagocytophilum* with the highest similarity to our haplotypes were identified from GenBank for phylogenetic analyses. Similarly sequences of the known human pathogen and nonpathogenic *A. phagocytophilum* were also selected from the GenBank for comparison in the phylogenetic tree. Phylogenetic analysis was carried out and phylogenetic tree was constructed using the maximum likelihood analyses in the MEGA 11 software using Kimura 2-parameter model and consensus tree inferred at 1000 bootstrap replications.

Chi-square tests performed in SPPSS v 20 statistical software were used to assess the statistical variation in infection prevalence among populations and across hosts at 95% confidence intervals.

## 3. Results

### 3.1. PCR-HRM Analysis of Blood and Tick Samples

**Figure 2:**
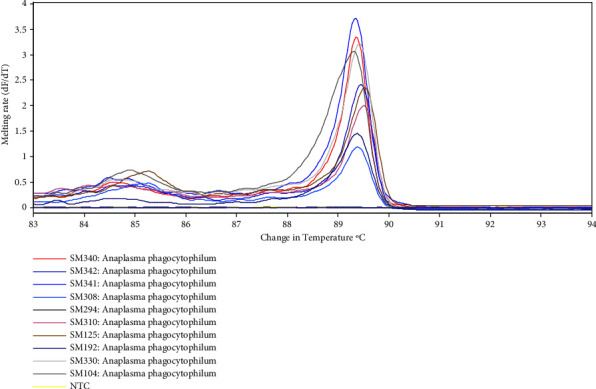
Typical representation of HRM profile plot of *Anaplasma phagocytophilum* from small mammals in Laikipia County performed in a Rotor-gene Q thermocycler using 16S rRNA gene primers and analyzed by Rotor gene software. Sterile water was used as a negative test control.

A total of 19 out of 385 analyzed samples were positive for *A. phagocytophilum* using the 16S rRNA gene primers translating to an overall prevalence of 4.9%. Specific small mammal species found positive were *Helogale parvula, Arvicanthis niloticus, Acomys kempi, Saccostomus mearnsi, Aethomys hindei, Mastomys natalensis*, and *Mus* spp. as shown in Table 1. Prevalence of *A. phagocytophilum* varied among small mammal species (*P*=0.021) with highest prevalence detected in *A*. *niloticus* (18.8%). *A. phagocytophilum* was not detected in *Acomys percivali, Crocidura allex, Dendromus insignis, Elephantulus rufescens, Gerbilliscus robustus, Grammomys dolichurus, Ichneumia albicauda, Myomyscus brockmani, Xerus erythropus, Rattus rattus*, and *Lemniscomys striatus. H*. *parvula* recorded a percentage positivity of 50% due to few number samples that were collected and screened. The HRM profile plots of the amplified *A. phagocytophilum* from blood samples were illustrated as change in fluorescence thresholds (dFT/dT) displayed against temperature (C) as shown in Figure 2.

**Figure 3:**
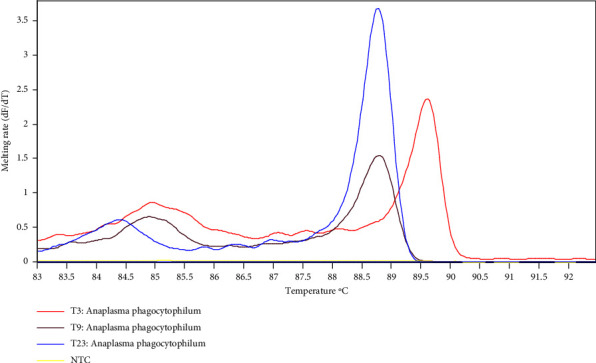
Typical representation of HRM profile plot of *Anaplasma phagocytophilum* amplified from ticks associated with small mammals in Laikipia County performed in a Rotor-gene Q thermocycler using 16S rRNA gene primers and analyzed by Rotor gene software. T3, T9, and T23 denoted different *Anaplasma phagocytophilum* haplotypes.

A total of 84 tick samples were identified as *Ornithodoros savignyi* (54/84) (64%), *Haemaphysalis leachi* (27/84) (32%), *Hyalomma truncatum* (2/84) (2%), and *Amblyomma* sp. (1%). The amplification of the tick samples using 16S rRNA species specific primers resulted into 3 positive samples (3/84) equivalent to a prevalence of 3.6%. The *H*. *leachi* species prevalence was 11.1%. *O*. *savignyi, H*. *truncatum*, and *Amblyomma* sp. tested negative for *A. phagocytophilum* using the 16S rRNA primers.  Figure 3 illustrates the HRM profile plots of amplified *A. phagocytophilum* from tick samples showing a change in fluorescence thresholds (dFT/dT) displayed against temperature (C).

### 3.2. Sequencing and Phylogenetic Analysis

**Figure 4:**
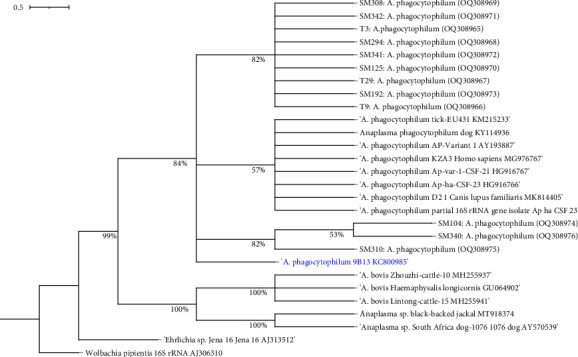
Phylogenetic relationship of *Anaplasma phagocytophilum* haplotypes in blood and tick samples from small mammals in Laikipia County, Kenya, with representatives from other regions. Arrow shows haplotypes from Kenya reported in this study.

Sequence results and the GenBank BLAST analysis of the 16S rRNA haplotypes from this study showed a similarity of 98% to 100% to a previously documented isolate 9B13 from *Alces alces* (KC800985). Sequence alignment of *A. phagocytophilum* haplotype sequences in this study showed 98–100% similarity, whereas haplotypes SM 310, SM 104, and SM 340 revealed nucleotide polymorphisms at locations 35 and 493; SM 310 and SM 340 at position 183, 205 and 370; and SM 308 at position 62. Phylogenetic analysis of the *A. phagocytophilum* haplotypes sequences revealed that haplotypes in this study were clustered in the two distinctive clades and shared a common ancestry with *A. phagocytophilum* isolate 9B13 from *Alces alces*, the closest GenBank blast match with 84% bootstrap support as shown in Figure 4. The nonpathogenic variant 1 of the *A. phagocytophilum* extracted from GenBank was clustered together forming a distinctive clade with 57% bootstrap support. The trees were edited by Interactive tree of life software accessed through https://itol.embl.de. *Wolbachia pipientis* partial 16S rRNA gene, strain trk1/dsz isolated from *Trachelipus rathkii* (AJ306310), was employed as the outgroup. All the haplotypes were submitted and registered in GenBank under the accession numbers OQ308965–OQ308976.

## 4. Discussion

This study involved molecular detection of *A. phagocytophilum* in wildlife small mammals and their infesting ticks from wildlife-livestock shared habitats in Laikipia County. Such a distinct human-livestock-wildlife ecology facilitates the persistence of numerous new zoonotic pathogens, which is exacerbated by the increase in anthropogenic activities [24]. It is documented that small mammals (rodents) in these ecological habitats are important epidemiological factors in the tick-borne pathogen dynamics and emergence including the *Anaplasma* species [19, 25, 26]. Small mammal species are involved in the transmission and maintenance of tick-borne pathogens in these interfaces [27–30].

The analysis of the 16S rRNA indicated an overall prevalence of 4.9% in small mammals and 3.6% in the infesting ticks. While *A. phagocytophilum* is considered widely distributed pathogen found in various small mammal species [6], the prevalence in this study was between 0 and 18.7% in specific small mammal species with overall prevalence of 4.9%. This prevalence is slightly higher compared to studies that reported *A. phagocytophilum* from Central Europe with a prevalence ranging from 1.6 to 2.2% [31] and Italy with a prevalence of 2.5% [32], and similarly, 5.4% prevalence was reported by Rosso et al. [11].

*Anaplasma phagocytophilum* have also been documented in various small mammals such as *Apodemus* spp., *Sorex minutus, Myodes* spp., *Rattus rattus,Microtus* spp. *Crocidura* spp., *Erinaceus europaeus*, and *Hystrix cristata* with prevalence ranging from 1 to 15% [32–34] which is in range with the prevalence reported in this study. The diversity of possible hosts, which is impacted by both climatic and environmental variables, might be one explanation for the variations in frequency between studies [32]. The molecular detection protocol that employs amplification of the conserved region of the 16S ribosomal RNA gene has been utilized successfully in detection of various *Anaplasma* species. A study conducted in small mammals found in localities surrounding US military installations in Korea by molecular amplification of the 16S rRNA reported a prevalence of 5% which is similar to the prevalence of *A. phagocytophilum* reported in this study [35].

In African countries, there are limited reports on the distribution and prevalence of *A. phagocytophilum* given the broad range of small mammal species present in the ecosystem. Nevertheless, various studies have documented *A. phagocytophilum* in domesticated ruminants, canines, wild carnivores, wild ungulates species, wild primates, and their related ectoparasites in ecosystem shared with small rodent mammals [36–38]. In a recent study done in South Africa, microbiome sequencing confirmed the presence of *A. phagocytophilum* in the blood from *Mastomys natalensis* and *Rattus tanezumi* [39] sampled from human settlements. In Kenya, *A. phagocytophilum* was detected in nonhuman primates [40] and *Rhipicephalus maculatus* [28] found in human-wildlife interfaces in Laikipia and Shimba Hills in Kenya, respectively. The close proximity and existence of these rodents in the reported ecosystem indicates the risk of rodents acting as reservoir host for *A. phagocytophilum.*

This study also reports the presence of *A. phagocytophilum* in *Haemaphysalis leachi* ticks through amplification and sequencing of the 16S rRNA gene primers. *A. phagocytophilum* has been extensively researched and explored in ticks mostly in the northern hemisphere and Asian countries particularly in the *Ixodes* and *Rhipicephalus* species of ticks [41] and recently reported in *Rhipicephalus maculatus* from Shimba Hills in Kenya [28]. The study also illustrates a prevalence of 3.6% of *A. phagocytophilum* which is in range with other documented studies from other ticks across the world. In the United States of America, prevalence of <1% to 50% has been reported in *I. scapularis* and <1% to 10% in *I. pacificus* as reviewed by Stuen et al. [6]. A study conducted in Turkey also demonstrated the presence of *A. phagocytophilum* in *Ixodes ricinus* ticks with a prevalence of 2.7% in Istanbul and 17.5% in the Kirklareli area [42]. In Asian countries, the prevalence is variable depending on species of ticks involved although prevalence ranging from <1% to 21.6% was documented in *I. persulcatus* [43]. This study reports the detection of A. *phagocytophilum* in *H. leachi*. Vector competence of the tick species needs to be explored further. The molecular diagnostic techniques in the quantification of vector competence are still considered a limiting factor [22] although the diagnostic method is still a useful tool in the epidemiological studies for various pathogens including *A. phagocytophilum.*

Phylogenetic analysis of the *A. phagocytophilum* haplotypes from this study compared to other documented haplotypes revealed a close relationship with haplotypes from Europe, China, and Africa (South Africa) that were documented as nonpathogenic in *Canis familiaris*, *Alces alces, Apodemus agrarius*, and host ticks (*Haemaphysalis longicornis*). All haplotypes from this study were clustered together in two distinctive clades supporting the inference that *A. phagocytophilum* haplotypes in rodents and associated ticks circulate in single enzootic cycles. Further studies should be carried out to evaluate the pathogenicity of these circulating *A. phagocytophilum* species within the Laikipia ecosystem.

## 5. Conclusion and Recommendation

*Anaplasma phagocytophilum* is present in small rodent wildlife mammals in both the conservation areas and livestock grazing lands of Laikipia County, Kenya. *A. phagocytophilum* is also present in ticks infesting the small wildlife mammals. *A. phagocytophilum* in rodents and associated ticks belongs to a clade suggesting a single enzootic cycle. Although our study does not allow us to determine the zoonotic and pathogenic potential of *A. phagocytophilum*, further investigation should be carried out to determine the potential risk given the interaction between people and wildlife populations in such a shared habitat.

Further investigation on the presence of the *A. phagocytophilum* in other species of mammals and ticks is also recommended to determine the overall prevalence and establish the public health risk potential. Such knowledge is important in formulating policies for prevention of these emerging zoonotic diseases.

## Figures and Tables

**Table 1:** Prevalence of *Anaplasma phagocytophilum* in small rodent mammals in Laikipia County, Kenya.

Animal species	Common name	Tested (*n*)	*A. phagocytophilum* positivity, *n* (%)
*Acomys kempi*	Kemp's spiny mouse	44	6 (13.6)
*Acomys percivali*	Percival's spiny mouse	34	0 (0.00)
*Aethomys hindei*	Hinde's rock rat	32	2 (6.3)
*Arvicanthis niloticus*	The African grass rat	16	3 (18.8)
*Crocidura allex*	The East African highland shrew	7	0 (0)
*Dendromus insignis*	African climbing mouse	4	0 (0)
*Elephantulus rufescens*	Elephant shrew	2	0 (0)
*Gerbilliscus robustus*	Fringe-tailed gerbil	34	0 (0)
*Grammomys dolichurus*	Woodland thicket rat	3	0 (0)
*Helogale parvula*	The common dwarf mongoose	2	1 (50.0)
*Ichneumia albicauda*	The white-tailed mongoose	7	0 (0)
*Lemniscomys striatus*	Typical striped grass mouse	11	0 (0)
*Mastomys natalensis*	The common African rat	57	2 (3.5)
*Mus* spp.	Mice	58	1 (1.7)
*Myomyscus brockmani*	Brockman's rock mouse	5	0 (0)
*Rattus rattus*	Black rat	6	0 (0)
*Saccostomus mearnsi*	Mearns's pouched mouse	55	4 (7.3)
*Xerus erythropus*	The striped ground squirrel	8	0 (0)
Total		385	19

## Data Availability

The data used to support the findings of this study are available from the corresponding author upon reasonable request.
